# THE EFFECTS OF WISDOM AND HEALTH ON NORTH AMERICAN SENIORS’ WELL-BEING SINCE COVID-19

**DOI:** 10.1093/geroni/igad104.0395

**Published:** 2023-12-21

**Authors:** Zhe Feng, Michel Ferrari, Pouria Saffaran, Melanie Munroe, Asma Shamim, Stephanie Morris

**Affiliations:** University of Toronto, Toronto, Ontario, Canada; University of Toronto, Toronto, Ontario, Canada; University Of Toronto, Toronto, Ontario, Canada; Remedy Institute, Toronto, Ontario, Canada; University of Toronto, Toronto, Ontario, Canada; University of Toronto, Toronto, Ontario, Canada

## Abstract

Public health restrictions necessitated by COVID-19 resulted in significantly reduced social contact for many older people, given their increased risks of infection and developing severe symptoms, even death. While reduced social contact can be a major cause of distress, our recent North American study (n=307) found 3 levels of resilience to the impact of COVID-19 – high (“well-adapted”), average (“getting-by”) and low (“struggling”) – associated with changes to wellbeing before (Time1), in summer 2020 (Time2), and about 18 months after (Time3). The present study investigates the wellbeing trajectory of 69 older individuals (Mage = 58.83, SDage = 7.21, max. = 77, min. = 50) within that larger sample who reported closely following local physical distancing recommendations. Specifically, it examines how their wellbeing was affected by their country of residence, and self-reported personal wisdom, self-transcendence, and health at Time2 and Time3. Simple logistic regression models suggest that, across Time2 and Time3, higher wisdom and better health were associated with higher likelihoods of being well-adapted vs. just getting-by. Higher self-transcendence at Time3 but not Time2 increases the likelihood of being well-adapted. Multiple logistic regressions with country, personal wisdom, self-transcendence, and health as predictors show that, controlling for all other variables in the model, higher wisdom and better health at Time2, as well as higher self-transcendence and better health at Time3, increase the likelihood of being well-adapted vs. just getting-by. Our findings demonstrate the protective values of personal wisdom, self-transcendence, and health during prolonged periods of isolation and stress for the older population.

